# Author Correction: Improving 10-deacetylbaccatin III-10-β-*O*-acetyltransferase catalytic fitness for Taxol production

**DOI:** 10.1038/ncomms16221

**Published:** 2018-07-13

**Authors:** Bing-Juan Li, Hao Wang, Ting Gong, Jing-Jing Chen, Tian-Jiao Chen, Jin-Ling Yang, Ping Zhu

Nature Communications 8: Article number: 15544; DOI: 10.1038/ncomms15544 (2017); Published: 05 18 2017, Updated: 07 13 2018

In the originally published version of this Article, financial support was not fully acknowledged. The PDF and HTML versions of the Article have now been corrected to include support from the National Natural Science Foundation of China grant number 81573325.

