# Supplementary material for: Improving 10-deacetylbaccatin III-10-β-O-acetyltransferase catalytic fitness for Taxol production
Source: Nat Commun. 2017 May 18;8:15544. doi: 10.1038/ncomms15544 (PMC5454391; doi:10.1038/ncomms15544)
Supplement: Supplementary Information — Supplementary Figures, Supplementary Tables [file ncomms15544-s1.pdf]

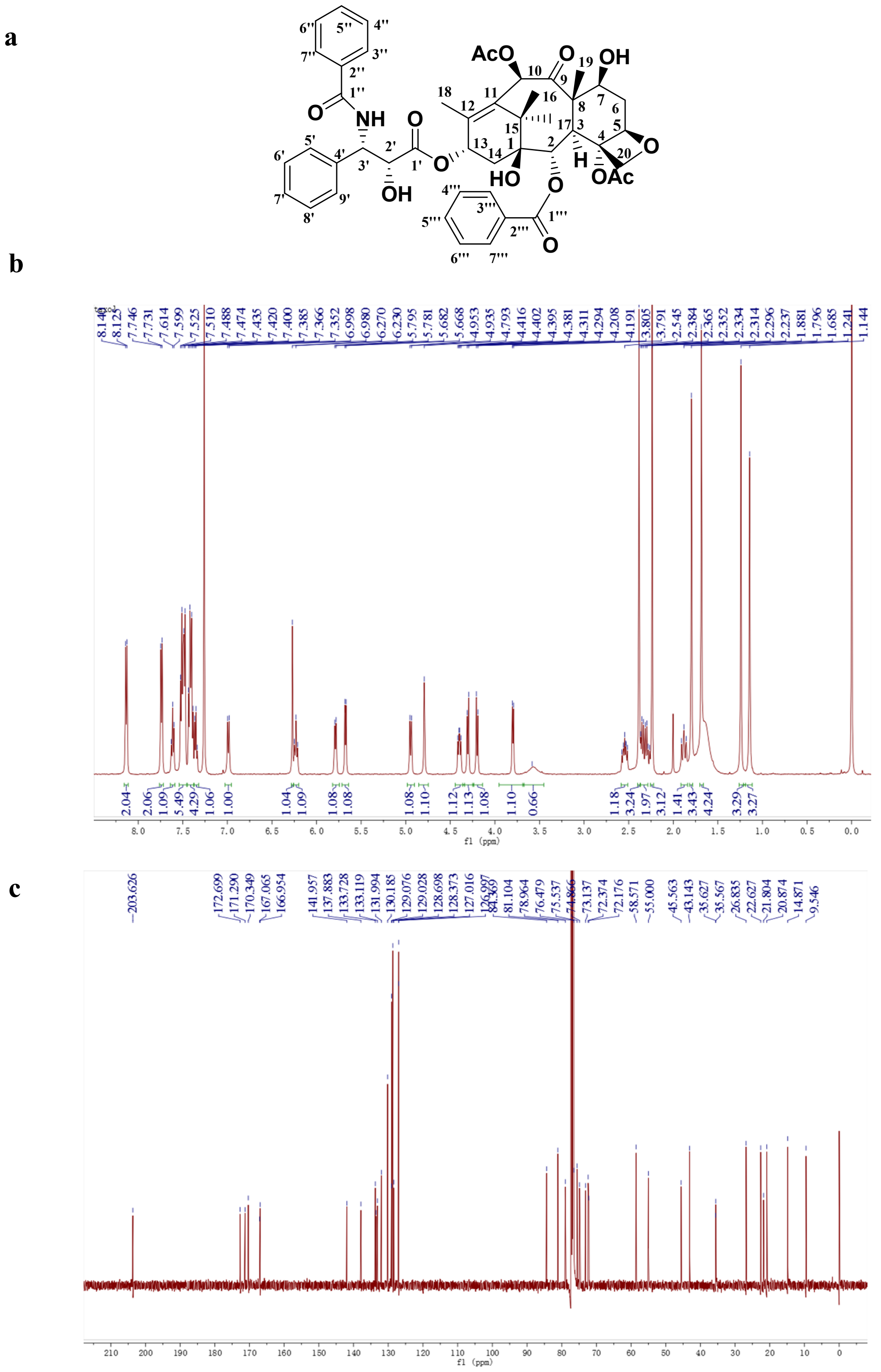

Supplementary Figure 1. The structure of Taxol and NMR spectra of the catalytic products of the recombinant DBAT (a.The structure of Taxol. b.The ^{1}H-NMR spectrum of product. c. The ^{13}C-NMR spectrum of product.). Taxol from the 50 ml reaction system was purified by HPLC then dissolved in $CDCl_3$ and confirmed by ^{1}H-NMR and ^{13}C-NMR analysis.

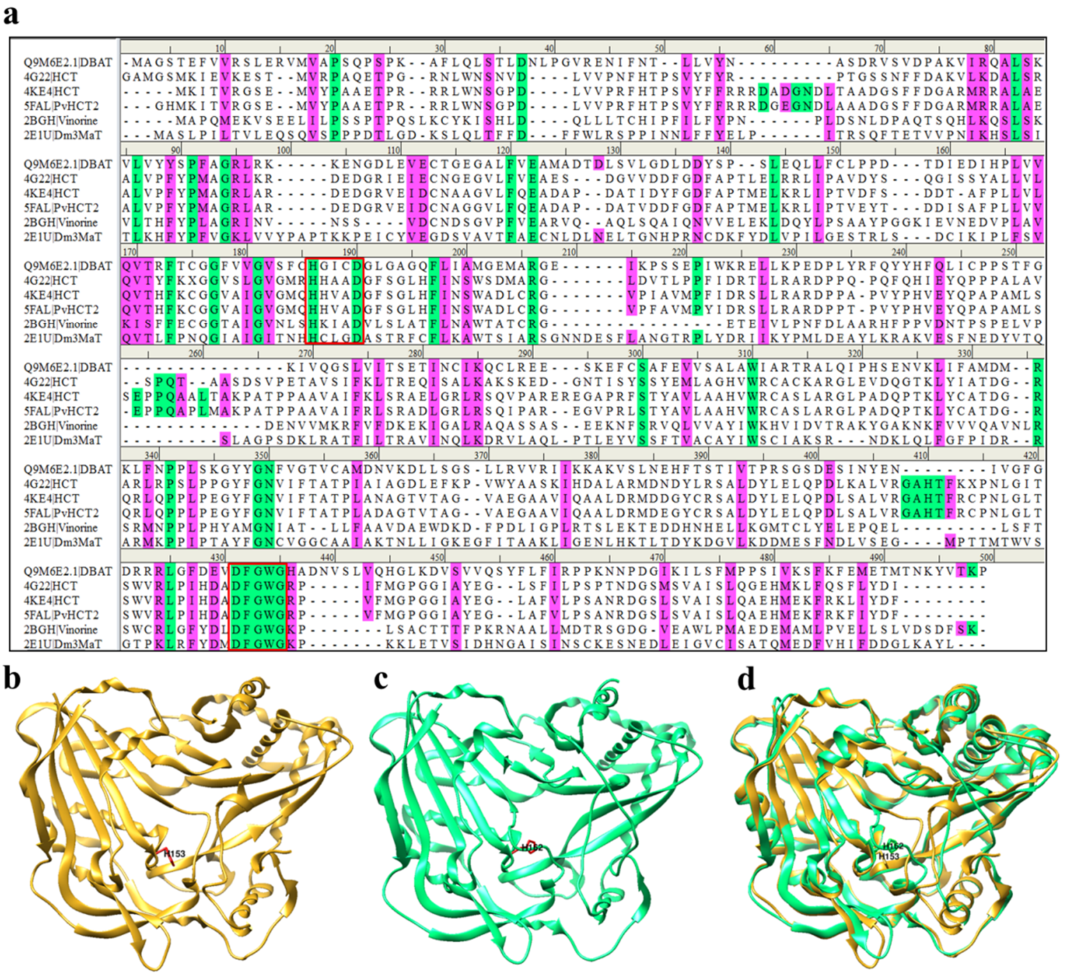

Supplementary Figure 2. Sequence and structure alignments of DBAT with other BAHD members. (a) Sequence alignment of DBAT with other BAHD members. The two conserved HXXXD and DFGWG motifs are marked in red boxes; the identical and similar residues are colored in green and pink, respectively. Accession numbers are as follows: DBAT, Q9M6E2.1; HCT, PDB: 4G22; HCT, PDB: 4KE4; PvHCT2, PDB: 5FAL; Vinorine synthase, PDB: 2BGH; Dm3MaT, PDB: 2E1U. (b) The structure of the HCT that was used as a template for DBAT homology modeling. (c) The structure of the predicted DBAT. (d) Structure alignment of DBAT with HCT (PDB: 4G22). HCT was colored in yellow, DBAT was colored in green.

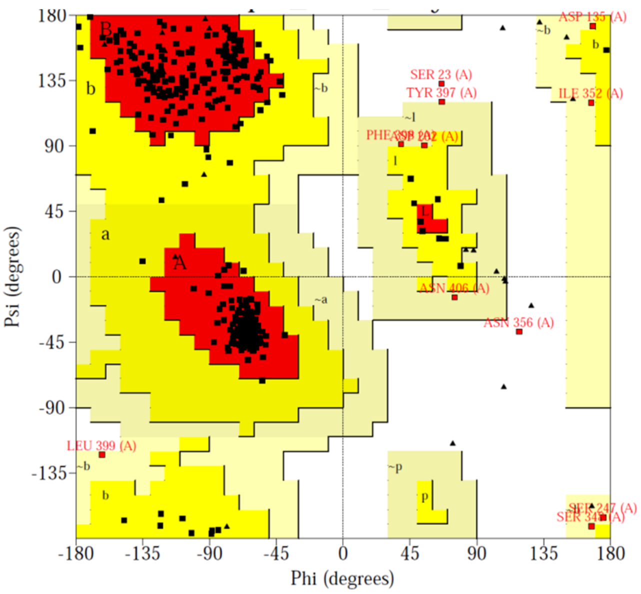

Supplementary Figure 3. Evaluation of the predicted structure of DBAT by Ramachandran plot.

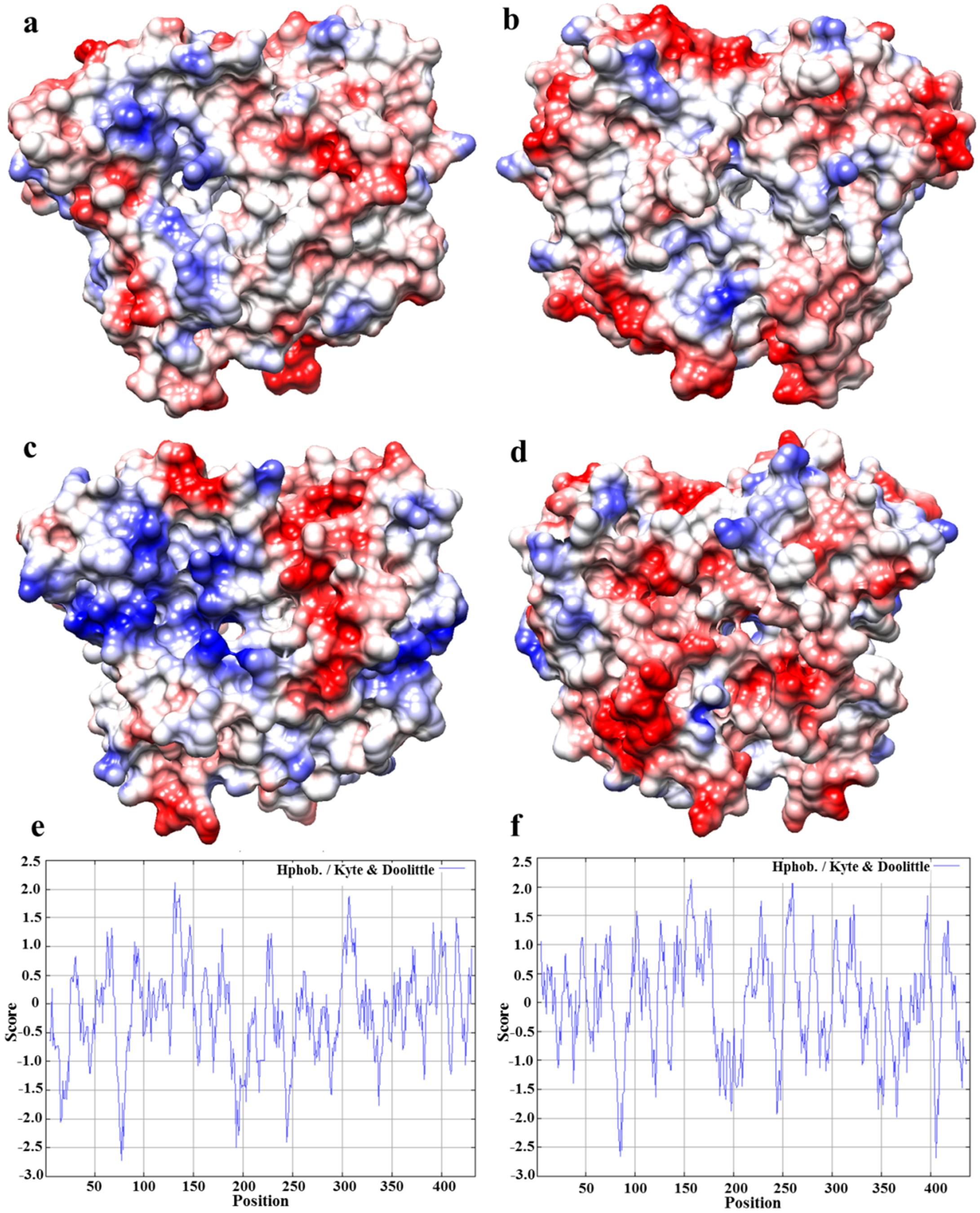

Supplementary Figure 4. Distribution of hydrophobicity and electronegativity residues in HCT and DBAT. (a) The front side of HCT. (b) The back side of HCT. (c) The front side of DBAT. (d) The back side of DBAT. (e) Electronegativity residue analysis of HCT. (f) Electronegativity residue analysis of DBAT. Residues were colored from red for negative potential, through white for near neutral, to blue for positive potential.

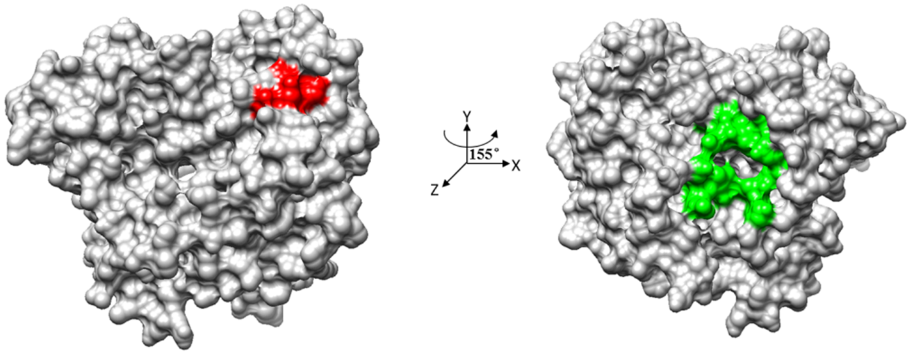

Supplementary Figure 5. The front side (left) and back side(right) of the predicted DBAT (DFGWG motif was colored in red, the predicted acyl acceptor substrate pocket was colored in green).

Supplementary Table 1. Comparison on the bioconversion of XDT to DT among different strains

Strains	Substrate Concentration (mg ml^{-1})	Reaction volume (ml)	XDT conversion rate (%)	DT yield (mg ml^{-1})	References
Moraxella sp.	0.25	2	~100	0.23	[23]
Leifsonia shinshuensis	0.5	2	~100	0.4	[24]
Enterobacter sp.	2.0	25	45	0.76	[25]
Cellulosimicrobium cellulans	0.5	100	91	0.3~0.4	[26]
Recombinant *Pichia pastoris*	15	1,000	~83	10.58	[32]
Recombinant *Pichia pastoris*	15*	10,000	~83	9.82**	[32]

*XDT_{ex} (containing 71.08% XDT, 10.67% 7-β-xylosyl-10-deacetylcepholamanine and 12.05% 7-β-xylosyl-10-deacetyltaxol C, w/w). **DT_{ex} (DT, 7.54; 10-deacetylcepholamanine, 1.05; 10-deacetyltaxol C, 1.23; mg ml^{-1}).

Supplementary Table 2. Specific activities of the six recombinant DBATs

Enzyme activity	*Taxus cuspidata*	*Taxus brevifolia*	*Taxus x media*	*Taxus canadensis*	*Taxus baccata*	*Taxus wallichiana var.*
（U mg^{-1}） （10-DAB）	206.6±6.7	214.9±15.6	198.7±7.5	41.5±8.2	1.1±0.4	215.7±8.5
（U mg^{-1}） $\times 10^{-1}$ （DT）	2.60±0.21	2.64±0.10	1.79±0.06	0.38±0.04	0.03±0.06	1.82±0.06

The data represent the means±s.d., *n*=3.

Supplementary Table 3. ^{1}H (500 MHz) and ^{13}C (125 MHz) NMR data for product ② ($CDCl_3$)

Position	δ_H,mult(J in Hz)	δ_C	Position	δ_H,mult(J in Hz)	δ_C
1		78.9	4-O-C*OCH$_3$		170.3
2	5.68,d (7.0)	74.9	4-O-COC*H$_3$	2.36,s	22.6
3	3.79,d (7.0)	45.6	10-O-C*OCH$_3$		171.3
4		81.1	10-O-COC*H$_3$	2.28,s	20.8
5	4.94,dd (9.0, 2.0)	84.3	1'		172.8
6	2.55, m, 1.88,m	35.6	2'	4.80, brs	73.1
7	4.40,dd (9.0, 6.0)	72.2	3'	5.78,d (9.0, 2.0)	55.0
8		58.6	4'		137.9
9		203.6	5'and9'	7.49,d (7.5)	127.0
10	6.27,s	75.5	6'and8'	7.43,t (7.5)[a]	128.7
11		133.1	7'	7.36,d (7.5)	128.4[a]
12		142.0	1''		167.1
13	6.23,dd	72.4	2''		133.5
14	2.31,m, 2.27,m	35.7	3''and7''	7.73,d (7.5)	127.1
15		43.2	4''and6''	7.42,t (7.5)[a]	129.0[a]
16	1.24,s	26.8	5''	7.51,d (7.5)[a]	131.9
17	1.14,s	21.8	1'''		167.0
18	1.80,s	14.8	2'''		128.4[a]
19	1.69,s	9.5	3'''and7'''	8.13,d (7.5)	130.2
20	4.30,d(10.5), 4.19,d (10.5)	76.5	4'''and6'''	7.52,t (7.5)[a]	129.1[a]
NH	6.98,d (9.0)		5'''	7.61,d (7.5)	133.7

[a]Overlapping signals.

Supplementary Table 4. Activities of DBAT and its mutants obtained by alanine scaning against 10-DAB and DT

	DT		10-DAB	
	U mg^{-1} ($\times10^{-2}$)	Relative activity(%)	U mg^{-1} ($\times10$)	Relative activity (%)
Con	26.00(±1.12)	100.00	20.66(±0.67)	100.00
S351A	25.45(±1.91)	97.87	19.56(±1.27)	94.69
G38A	37.69(±0.88) **	144.95	18.82(±0.51)	91.09
S396A	23.59(±1.32)	90.72	18.99(±0.48)	91.91
R40A	21.06(±0.79)	81.00	18.82(±0.96)	91.08
N353A	15.28(±0.44)	58.77	17.64(±0.22)	85.38
E41A	14.48(±0.07)	55.71	14.81(±0.46)	71.70
C165A	16.15(±0.26)	62.13	13.16(±0.36)	63.68
F160A	6.34(±0.07)	24.37	12.50(±0.09)	60.49
F301A	41.38(±1.12) **	159.17	9.75(±0.32)	47.18
P37A	2.78(±0.02)	10.71	8.00(±0.10)	38.72
F400A	1.32(±0.02)	5.07	3.58(±0.02)	17.32
F44A	0.22(±0.01)	0.85	1.89(±0.01)	9.15
G359A	2.84(±0.03)	10.92	1.67(±0.01)	8.06
I164A	0.19(±0.01)	0.72	0.25(±0.01)	1.20
G361A	ND	ND	ND	ND
R363A	ND	ND	ND	ND

The data represent the means±s.d., *n*=3. **P*<0.05 vs Control，***P*<0.01 vs Control (Student's *t*-test). ND: Not detected.

Supplementary Table 5. Activities of Gly[38] saturation mutants against DT

	U mg^{-1} (×10^{-2})	Relative activity (%)
DBAT	26.00(±1.12)	100.00
G38R	56.88(±3.11)**	218.77
G38S	54.42(±2.02)**	209.32
G38D	49.79(±1.76)**	191.52
G38H	47.01(±0.97)**	180.81
G38A	41.44(±2.98)**	159.38
G38N	28.40(±1.70)	109.21
G38T	24.70(±0.67)	95.01
G38P	22.37(±0.16)	86.04
G38M	21.62(±0.27)	83.16
G38Q	17.37(±1.10)	66.79
G38E	16.79(±0.08)	64.59
G38W	13.21(±0.09)	50.80
G38Y	11.10(±0.04)	42.68
G38V	11.02(±0.01)	42.37
G38C	10.68(±0.10)	41.07
G38I	9.99(±0.01)	38.42
G38L	7.71(±0.04)	29.66
G38F	5.67(±0.01)	21.81
G38K	ND	ND

The data represent the means±s.d., *n*=3. **P*<0.05 vs Control, ***P*<0.01 vs Control (Student's *t*-test). ND: Not detected.

Supplementary Table 6. Activities of Phe^{301} saturation mutants against DT

	U mg^{-1} ($\times 10^{-2}$)	Relative activity (%)
DBAT	26.00(±1.12)	100.00
F301V	74.10(±8.45)**	285.00
F301C	47.22(±3.42)**	181.60
F301A	41.60(±1.98)**	160.00
F301M	35.10(±1.95)*	135.00
F301L	33.18(±1.69)*	127.62
F301T	31.20(±1.52)*	120.00
F301S	30.06(±1.37)	115.62
F301Y	25.47(±1.02)	97.96
F301G	18.80(±0.54)	72.30
F301R	13.95(±0.31)	53.67
F301H	10.02(±0.15)	38.52
F301W	7.23(±0.08)	27.79
F301I	7.02(±0.09)	27.00
F301K	0.56(±0.02)	2.16
F301P	0.11(±0.02)	0.41
F301D	0.16(±0.01)	0.60
F301Q	0.10(±0.01)	0.40
F301E	0.16(±0.02)	0.60
F301N	0.13(±0.02)	0.50

The data represent the means±s.d., n=3. *P<0.05 vs Control, **P<0.01 vs Control (Student's t-test).

Supplementary Table 7.Yields of Taxol in the two enzyme coupled catalytic system

Volume	1 ml	10 ml	50 ml
Taxol yield（μg ml^{-1}）	657.32±13.15	637.24±5.10	635.35±6.27

The data represent the means±s.d., *n*=3.

Supplementary Table 8. Cytotoxicities of Taxol and its analogues against human cancer cells (MTT method)

Compounds	IC_{50} (μM)				
	HCT116	NCI-H460	MGC803	HepG2	MCF-7
Taxol	0.0109	0.0211	0.00772	0.0691	0.00930
DT	0.0780	0.482	0.0508	0.482	0.0972
XDT	0.476	3.22	0.491	5.42	3.81

Note: HepG2, human hepatocellular liver carcinoma cell line; MCF-7：human breast carcinoma cell line; NCI-H460：human lung carcinoma cell line; HCT116：human colon carcinoma cell line; MGC803：human stomach carcinoma cell line.

Supplementary Table 9. The gradient elution conditions for 10-DAB and baccatin III analysis

Time (min)	Acetonitrile (%)	Water (%)
0	28	72
15	40	60
30	40	60
33	100	0
43	100	0
46	28	72
51	28	72

Supplementary Table 10. The gradient elution conditions for XDT, DT and Taxol analysis

Time (min)	Acetonitrile (%)	Water (%)
0	28	72
15	40	60
16	44	56
26	44	56
36	48	52
38	100	0
48	100	0
49	28	72
59	28	72

Supplementary Table 11. The linear equations of baccatin III, DT and Taxol

	Linear equations	Correlation coefficient
Baccatin III	Y=43291X+35693	R^2=0.9999
DT	Y=66664X+175819	R^2=0.9995
Taxol	Y=148089X+41221	R^2=0.9996

Supplementary Table 12. Primer sequences. The primers were used for construction the alanine mutants and saturation mutants

Primers	Sequences (5′→3′, the corresponding mutant bases were labeled underlined)
37 AF	TGACAATCTAGCAGGGGTGAG
37 AR	CTCTCACCCCTGCTAGATTGT
38AF	GACAATCTACCAGCGGTGAGAG
38AR	TTTTCTCTCACCGCTGGTAGA
40AF	CCAGGGGTGGCAGAAAACATT
40AR	AAATGTTTTCTGCCACCCCTG
41AF	CAGGGGTGAGAGCAAACATTT
41AR	AAAATGTTTGCTCTCACCCCTG
44AF	AGAAAACATTGCTAACACCTTG
44AR	AAGGTGTTAGCAATGTTTTCT
160AF	GGGTGAGTGCCTGCCATGGTATATG
160AR	CCATGGCAGGCACTCACCCCTAC
162AF	TTTCTGCGCTGGTATATGTGATG
162AR	CATATACCAGCGCAGAAACTCACC
164AF	CCATGGTGCATGTGATGGACTAG
164AR	CATCACATGCACCATGGCAGAAAC
165AF	CATGGTATAGCTGATGGACTAGG
165AR	AGTCCATCAGCTATACCATGGCAG
301A F	GGATACTACGGTAATGCTGTTGG
301A R	ATACGGTACCAACAGCATTACC
351AF	TCAGATGAGGCTATCAATTATG
351AR	TAATTGATAGCCTCATCTGATC
353AF	TGAGAGTATCGCTTATGAAAAC
353AR	TGTTTTCATAAGCGATACTCTC
359AF	AACATAGTTGCATTTGGTGAT
359AR	CACCAAATGCAACTATGTTTTCA
361AF	GTTGGATTTGCTGATCGAAG
361AR	CCTTCGATCAGCAAATCCAACT
363AF	TTTGGTGATGCAAGGCGATTG
363AR	AATCGCCTTGCATCACCAAATC
396AF	AGTCGTGCAAGCTTATTTTCTTTTC
396AR	GAAAATAAGCTTGCACGACTGAAAC
400AF	TATTTTCTTGCCATACGACCTCC
400AR	GAGGTCGTATGGCAAGAAAATAAC
38NF	TACCANNKGTGAGAGAAAACATT
38NR	TCTCTCACMNNTGGTAGATTGTC
301NF	CGGTAATNNKGTTGGTACCGTATG
301NR	GTACCAACMNNATTACCGTAGTAT
